# Targeting at the Nanoscale: A Novel S-Layer Fusion Protein Enabling Controlled Immobilization of Biotinylated Molecules

**DOI:** 10.3390/nano6110199

**Published:** 2016-11-04

**Authors:** Melinda Varga

**Affiliations:** Electronics Packaging Laboratory, Department of Electrical Engineering and Information Technology, Technische Universität Dresden, Dresden 01069, Germany; varga@avt.et.tu-dresden.de; Tel.: +49-351-4633-6107; Fax: +49-351-4633-7035

**Keywords:** surface layer protein, *Sporosarcina ureae* ATCC 13881, quantum dots, self-assembly, biotinylated molecules

## Abstract

With the aim of constructing an S-layer fusion protein that combines both excellent self-assembly and specific ligand i.e., biotin binding ability, streptavidin (aa 16-133) was fused to the S-layer protein of *Sporosarcina ureae* ATCC 13881 (SslA) devoid of its N-terminal 341 and C-terminal 172 amino acids. The genetically engineered chimeric protein could be successfully produced in *E. coli*, isolated, and purified via Ni affinity chromatography. In vitro recrystallisation experiments performed with the purified chimeric protein in solution and on a silicon wafer have demonstrated that fusion of the streptavidin domain does not interfere with the self-assembling properties of the S-layer part. The chimeric protein self-assembled into multilayers. More importantly, the streptavidin domain retained its full biotin-binding ability, a fact evidenced by experiments in which biotinylated quantum dots were coupled to the fusion protein monomers and adsorbed onto the in vitro recrystallised fusion protein template. In this way, this S-layer fusion protein can serve as a functional template for the controlled immobilization of biotinylated and biologically active molecules.

## 1. Introduction

Biomolecular templating is fundamental in the development of advanced biosensors, bioreactors, affinity chromatographic separation materials, and many diagnostics such as those used in cancer therapeutics [1,2,3]. In this regard, biomolecules are used to precisely position nanoscale materials onto substrates. Since single molecules are by far too small to direct the formation of (supramolecular) complex shapes and patterns, the ordered structures of biological molecules and assemblies are excellent templates for constructing inorganic nanostructures and devices. In addition, genetic control over the template properties and coupling could bring to the forefront new platforms and material functionalities that will advance both biotechnology and nanoscale engineering.

Bacterial cell surface layers (S-layers) are two dimensional protein lattices that cover the cell surface of many Bacteria and Archaea [4]. These lattices are composed of identical (glyco)protein subunits, exhibit oblique (p1, p2), square (p4), or hexagonal (p3, p6) symmetry and are very porous. Due to the monomolecular structure of the lattices, the pores are identical in size and morphology, ranging between 2 and 8 nm [5].

The conditions required for extraction and disintegration have shown that S-layers are held together and onto the supporting envelope by non-covalent forces including hydrogen or ionic bonds, and hydrophobic or electrostatic interactions [6,7,8,9]. Isolated S-layer subunits maintain this inherent property of self-assembling in protein lattices. After detachment from the cell surface and disruption into monomers using chaotropic (e.g., guadinium hydroclorid or urea in the case of Gram-positive bacteria) agents, S-layers are able to reassemble into regular lattices, identical to those observed on intact cells upon removal of the chemical [10,11]. Reassembly can occur in solution, on solid supports such as silicon wafers, gold chips, and glass, on lipid films, and at the air/water interface [12]. The property of regular structure formation as well as the metal binding properties of S-layers have been considered for bionanotechnological applications [13]. Moreover, their utilization can be extended by the design of functional recombinant S-layer fusion proteins. By genetic modifications, S-layers have been given specific functions. For instance, an S-layer fusion protein consisting of the S-layer protein SbpA and a synthetic analogue of the B-domain of protein A capable of binding the fragment crystallisable (Fc) part of immunoglobulin G (IgG) was constructed and used for coating biocompatible microbeads for generating a specific adsorbent, which should find clinical application in the treatment of various autoimmune diseases [14]. Furthermore, for the treatment of IgE-mediated allergies by construction of vaccines, Bet v1 allergen was fused to the S-layer protein SbsC of *Bacillus stearothermophylus* ATCC 12980 and the fusion protein proved to be fully functional [15].

The mature S-layer protein SslA of *Sporosarcina ureae* ATCC 13881 is processed from a 1097 amino acid (aa) long precursor by the cleavage of a 30 aa signal peptide and self-assembles into protein lattices that exhibit square (p4) symmetry with a lattice constant of 12.2 nm [16,17]. The S-layer has a complex pattern of pores and gaps that are approximately 2 nm wide [18]. In vitro, the recombinant mature SslA self-assembles into micrometer sized, crystalline monolayers on silicon wafers, while in solution not only sheets but tube like structures have also been observed [17]. With various truncated SslA forms cloned, expressed, and purified from *E. coli*, it could be demonstrated that the central SslA domain of this S-layer protein is self-sufficient for the self-assembly [17]. In a previous study, a fusion protein consisting of streptavidin (aa 16-133) and the central SslA part (aa 341-925) was briefly presented [19]. In this work the cloning and expression of this chimeric protein (SslA_341-925_streptavidin) is described in detail and the protein is further characterized with respect to its self-assembling properties in solution and on a silicon wafer. Moreover, the electrophoretic mobility shift assay (EMSA) and transmission electron microscopy (TEM) are used to prove the accessibility of the streptavidin domain in the fusion protein lattice.

## 2. Results

### 2.1. Cloning and Expression of the SslA_341-925_streptavidin Fusion Protein

The DNA encoding the SslA_341-925_streptavidin fusion protein was created by overlap extension polymerase chain reaction (PCR). The chimeric gene was finally cloned into the pET46 Ek/Lic vector using the Ek/Lic strategy. The gene encoding SslA_341-925_streptavidin consists of 2160 bps and translates into a protein of 719 amino acids with a molecular weight of 76.5 kDa and a pI of 5.36. The final protein structure contains an N-terminal His_6_ tag.

**Figure 1:**
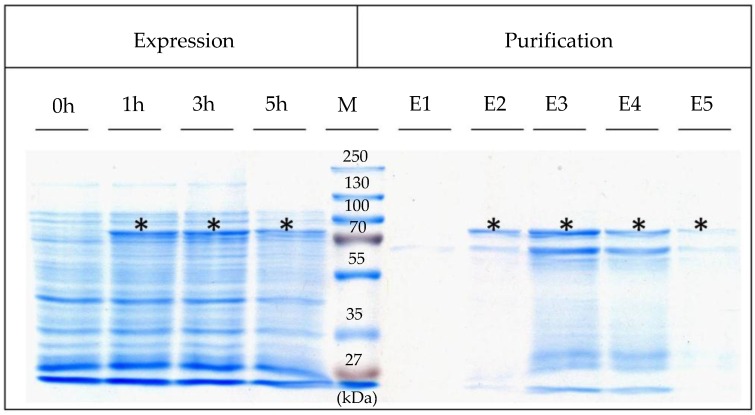
The Coomasie stained protein gel shows expression of SslA_341-925_streptavidin in *E. coli* Rosetta Blue (DE3) whole cell lysates after 0, 1, 3 and 5 h of induction with 1 mM IPTG. E1–E5 mark the elution fractions of the Ni affinity chromatography. M-protein ladder (Page Ruler™ Plus Prestained). 20 μg recombinant protein was loaded on the gel and its position is marked with star.

For heterologous expression of the chimeric gene, pET46Ek/Lic-SslA_341-925_streptavidin plasmids from positive clones were transformed into *E. coli* Rosetta Blue (DE3) cells. Recombinant gene expression was induced with IPTG (1 mM final concentration) and carried out at 30 °C for 5 h. Culture samples were harvested before and 1, 3 and 5 h after induction and subjected to sodium dodecyl sulfate polyacrylamide gel electrophoresis (SDS-PAGE) analysis. Expression of the chimeric gene encoding SslA_341-925_streptavidin was indicated by the appearance of an additional protein band with an apparent molecular mass of 76,000 Da on the SDS gel (Figure 1 Lane 2, 3, and 4 marked with star). The expression was however, moderate.

SslA_341-925_streptavidin was isolated from the soluble fraction of lysed *E. coli* cells and purified via Ni affinity chromatography. This purification method was chosen because the protein is expressed with an N-terminally encoded His_6_ tag. Fractions containing the purified protein were pooled, dialysed against purified water for 12 h at 4 °C, and analysed by SDS-PAGE. Results of the purification are presented in Figure 1, Lane 7, 8, 9, and 10 marked with star. The protein eluted at the expected molecular mass (76 kDa) indicating that is mainly monomeric (Figure 1 E2, E3, E4, E5). The additional protein band observed might represent degradation products in these lanes. The purification procedure yielded about 20 mg pure protein from a 3 L cell culture. The purified protein was stored at 4 °C until use. For further experiments i.e., in vitro recrystallisation, the protein from the last elution fraction was used since this was the most pure one.

### 2.2. Investigation of Self-Assembly Properties of the SslA_341-925_streptavidin Protein

After expression and purification, the self-assembling capability of the SslA_341-925_streptavidin fusion protein was tested by carrying out in vitro recrystallisation experiments in solution and on a functionalised silicon wafer.

**Figure 2:**
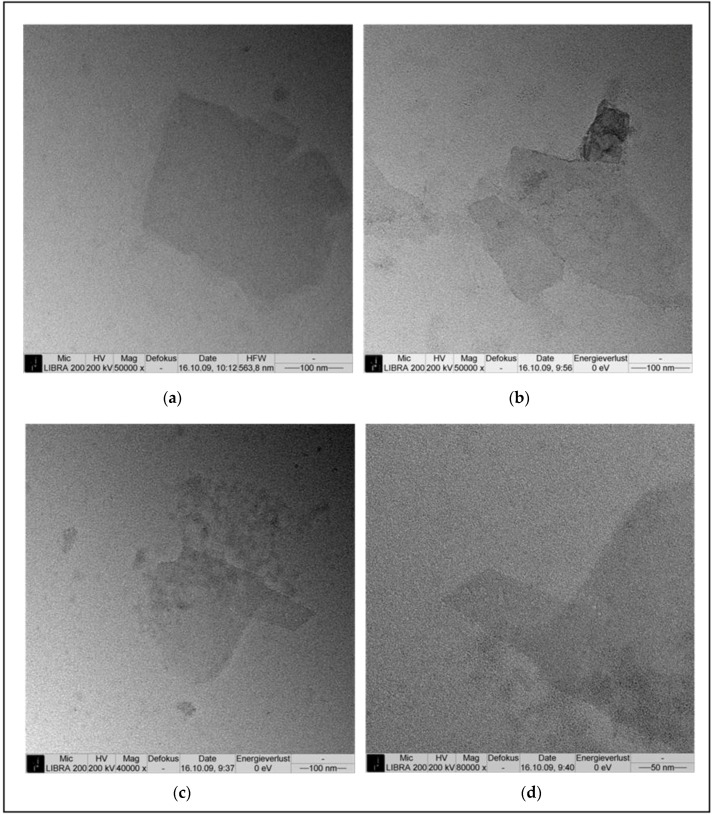
Transmission electron microscopy (TEM) micrograph showing the self-assembly of the SslA_341-925_streptavidin fusion protein in solution (**a**) In vitro recrystallisation resulted in mono- and multi-layered square shaped protein sheets; (**b**) The SslA_341-925_streptavidin fusion protein layers are polycrystalline consisting of individual patches that have grown until reaching neighboring patch edges; (**c**,**d**) Folding of the protein sheets occurs, giving rise to tubular structures.

To initiate the self-assembly process in solution, the purified fusion protein (at a protein concentration of 1 mg/mL) was first disrupted into monomers using 5 M GuHCl. After removal of the chemical by dialysis followed by a centrifugation step, the resulting monomer solution was diluted to 0.1 mg/mL with 0.1 mM CaCl_2_ and 0.5 mM Tris/HCl (pH = 9) and allowed to recrystallise for 48 h. Transmission electron microscopy (TEM) revealed that the SslA_341-925_streptavidin fusion protein was able to self-assemble in suspension into crystalline chimeric S-layer sheets (Figure 2). Square shaped coherent monolayers were formed, which had a size of approximately 200–250 nm, however multi-layered structures could also be recognized (Figure 2a). The square like edges of the protein layer resembles the structure and shape of the authentic S-layer form. Moreover, the self-assembled structures generated are polycrystalline (Figure 2b). Folding of the protein layers, a common feature among S-layers was also observed (Figure 2c,d).

**Figure 3:**
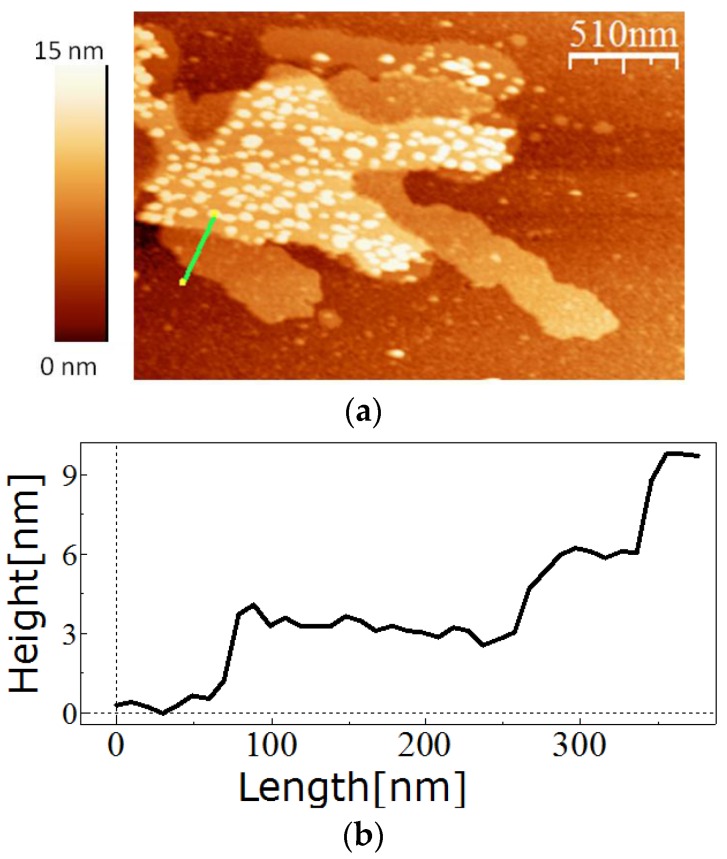
Self-assembly of the SslA_341-925_streptavidin on a silicon wafer. (**a**) The SslA_341-925_streptavidin fusion protein was recrystallised in vitro at pH = 3 in the presence of an APTES-functionalised silicon wafer (**b**) The cross-section profile along the green line shows the height of the protein layers (3.5 nm each).

The self-assembly properties of SslA_341-925_streptavidin on a solid substrate were also investigated. To this end, the purified protein was at first disintegrated into monomers with 5 M GuHCl, and then the monomers were allowed to recrystallise during a dialysis step against Tris/HCl pH = 3 on aminopropyltriethoxysilane (APTES)-treated silicon wafers. As shown in Figure 3, SslA_341-925_streptavidin self-assembled into crystalline, mono- and multilayered protein sheets on the silicon surface. The atomic force microscopy (AFM) topographical images indicate that the thickness of one monolayer is ~3.5 nm (Figure 3). The monolayers have a size between 500 and 700 nm while the top layers are relatively smaller. White dots observed in the AFM image can be considered nucleation points or small monomer clusters that will give rise to the next protein layer. The square lattice symmetry and lattice parameters of the chimeric protein could not be determined by Fast Fourier transformation analysis of the AFM micrographs. The reason could be the following: the native SslA protein possesses pores that are 2 nm wide; the wild-type streptavidin molecule has a height of 2 nm and a diameter of 11 nm. For fusion, only a fragment of the streptavidin was used, however this might still be large enough to cover the pores of the SslA_341-925_streptavidin fusion protein. Hence, the lattice periodicity cannot be observed.

### 2.3. Investigation of the Biotin Binding Properties of the SslA_341-925_streptavidin Fusion Protein

**Figure 4:**
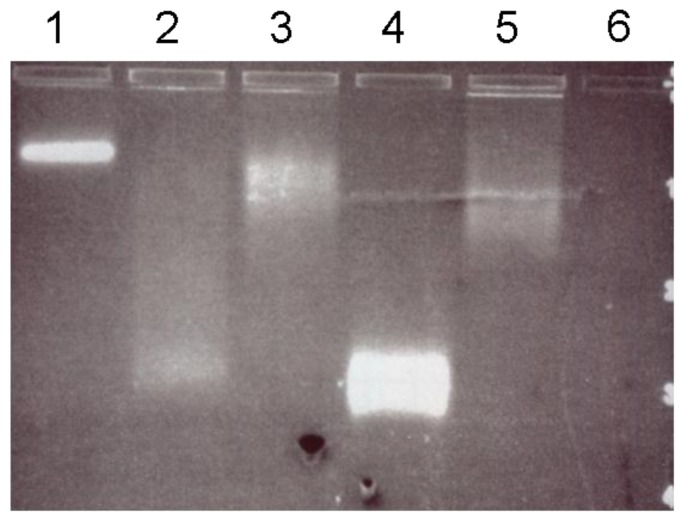
Agarose gel electrophoresis of QD-b and SslA_341-925_streptavidin mixtures at different ratios (QD-b/SslA_341-925_streptavidin in lane 1–3 was 1/10, 1/3, and 1/1 separately); single QD-b (lane 4); wild- type streptavidin/QD-b (lane 5) and SslA_341-925_streptavidin (lane 6).

For gaining evidence about the functionality of the integrated streptavidin domain, experiments were performed using biotinylated quantum dots (QD-b). The electrophoretic mobility shift assay (EMSA) method confirmed the coupling of QD-b to the SslA_341-925_streptavidin monomers (Figure 4).

Both QD-b and fusion protein molecules entered the gel and migrated towards the positive pole. When mixed (Figure 4, lane 1–3), a much slower running behaviour was observed in comparison to single QD-b molecules (Figure 4, lane 4), certifying the binding between the QD-b and SslA_341-925_streptavidin protein monomers. Migration in the gel was found to be strongly dependent on the protein concentration. It increased by lowering the amount of protein monomers in the mixture and the phenomenon took the appearance of a smear (spreading of the QD-b molecules according to how many protein molecules they bind) (Figure 4, lane 2 and 3). As a positive control, wild-type streptavidin was also incubated with QD-b (Figure 4, lane 5). Migration showed a similar smear and a slower migration pattern in comparison to single QD-b. The fusion protein (SslA_341-925_streptavidin) cannot be visualized under UV light; therefore lane 6 in Figure 4 does not show any visible band (negative control).

Additionally, it was observed that KHPO_4_ enhanced the conjugation process. As a buffer for incubating QD-b with the fusion protein, it has been much more efficient than Tris/HCl (pH = 9). GuHCl interferes with the gel electrophoresis, therefore in the preparation of the monomer solution, a dialysis step was inevitable. After dialysis, a centrifugation step ensured that only fusion protein (SslA_341-925_streptavidin) monomers were mixed with QD-b molecules.

**Figure 5:**
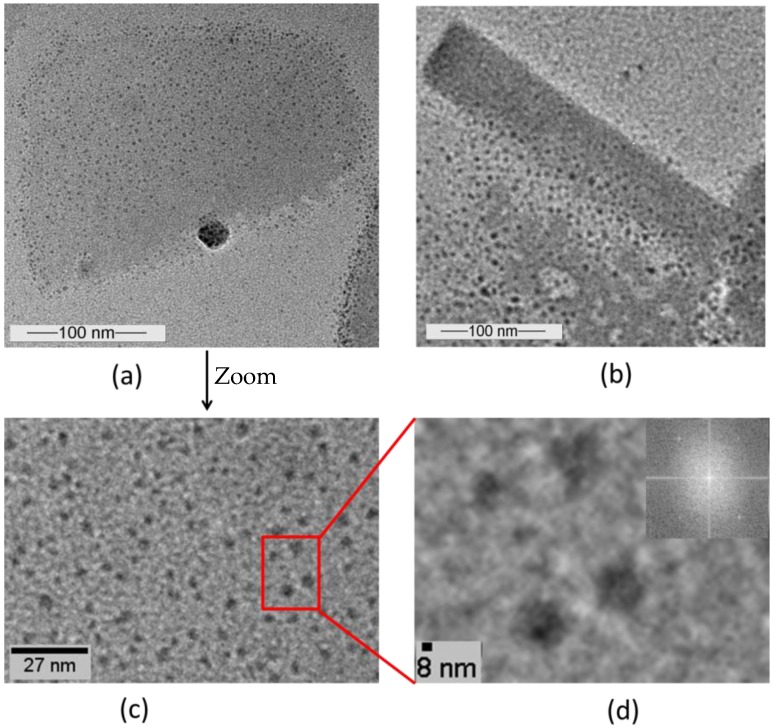
TEM micrographs showing biotemplating of biotinylated quantum dots (QD-b) onto the SslA_341-925_streptavidin fusion protein template. (**a**) The in vitro recrystallised SslA_341-925_streptavidin fusion protein layers were immobilised onto plasma treated C-Cu grids followed by the adsorption of biotinylated quantum dots (black dots) onto these layers. The particles are more abundant on the protein template; (**b**) On the tubular SslA_341-925_streptavidin structures marginal deposition of QD-b occurred; (**c**) Zoom in of image (**a**); (**d**) Zoom in of image (**c**) showing the adsorption pattern of quantum dots onto the SslA_341-925_streptavidin fusion protein template with fast Fourier transform FFT analysis.

In the next step, it was tested whether the streptavidin moiety is sterically accessible in the fusion protein lattices for binding biotinylated targets. To this end, the fusion protein was in vitro recrystallised in solution and afterwards immobilised onto plasma treated C-Cu grids. (The hydrophilic character of the grid directed the adsorption of the protein with its hydrophilic side). QD-b molecules were allowed to adsorb onto the chimeric protein sheets for 1 h. TEM micrographs shown in Figure 5 indicate that QD-b molecules, observed as black dots on the images, have bound onto the fusion protein template as they are more evident and abundant herein than on the background areas where the S-layers are physically absent (Figure 5a). These facts clearly show that the streptavidin domain remains exposed in the chimeric protein lattice and that self-assembly did not cause a steric hindrance for biotin binding. However, when the protein lattice folds into tube like structures, the streptavidin domain becomes buried and QD-b particles are observed only on the edges of the protein tubes (Figure 5b).

## 3. Discussion

Recombinant fusion proteins incorporating functional domains of distinct proteins could be relevant not only in drug development, but also in biotechnological applications such as the bio-functionalization of surfaces. The ability of isolated bacterial S-layer protein subunits to self-assemble into protein layers on solid surfaces makes them an almost ideal biological template for bio(nano)patterning. In this study an S-layer fusion protein that combines the remarkable self-assembling property of the S-layer moiety with the ligand binding function i.e., biotin binding function, of the streptavidin molecule is described and characterized.

In the design of this fusion protein, an important prerequisite was the preserved self-assembly potential of the S-layer part. A previous study about this S-layer protein showed that the central part of SslA (aa 341-925) is sufficient for in vitro self-assembly into monolayers exhibiting the p4 lattice structure [17]. Furthermore, the insertion of the functional domain has to be done at favourable positions in the polypeptide chain so that the whole fusion protein preserves the self-assembling ability and presents the additional function of the fused protein domain. The position for a functional fusion was selected based on the results of previous studies showing that usually the C-terminus of S-layers can be modified without affecting the protein properties and function, and on the fact that SslA despite the lack of its C-terminal part, has a preserved self-assembly potential [20]. In this context, streptavidin was fused to the C-terminus of the SslA_341-925_. Fusion of streptavidin to a truncated SslA version—in this case to the central part of SslA—constitutes an advantage over the fusion to the whole SslA sequence because it is shorter and in this sense, the chance that the fusion protein will be correctly folded is higher. Previous studies have shown that residues 16-133 of streptavidin are enough for biotin binding [21]. For fusion, this minimal sized form of streptavidin, that still retained the full biotin-binding activity, was used.

The SslA_341-925_streptavidin fusion protein could be stably expressed in *E. coli* and isolated from the soluble fraction of these cells. In the literature it was reported that streptavidin fused to the C-terminal of the S-layer protein of *Geobacillus stearothermophilus* PV72/p2 (SbsB) accumulated in the soluble fraction as well [22]. Without the S-layer fusion partner, streptavidin was found in inclusion bodies in *E. coli* cells. The His_6_ tag on the N-terminal of the SslA_341-925_streptavidin allowed an easy purification of this protein via Ni affinity chromatography. In addition to the band corresponding to the fusion protein, another one was also detected after purification. This might represent a degradation product. Similar results have been reported by Posseckardt J. [23].

Similar to the wild-type or full length recombinant SslA, the SslA_341-925_streptavidin chimeric protein could self-assemble in solution and on functionalised silicon surfaces demonstrating that fusion of the streptavidin domain does not interfere with the self-assembling properties of this S-layer. The monitored self-assembly products had the form of multi-layered sheets or monolayer cylinders.

Since the streptavidin domain used for fusion descended from wild-type streptavidin, it was assumed to show the typical high affinity for biotin. However, it remained to be investigated whether the biotin binding sites remain exposed and sterically available after the fusion or if they are blocked by the S-layer partner. In the mobility assay (gel electrophoresis technique) the interaction between SslA_341-925_streptavidin protein monomers and biotinylated quantum dots was monitored by applying a constant electric field and following the diffusion of the charged molecules in the porous gel matrix. Interestingly, the quantum dot/SslA_341-925_streptavidin fusion protein complexes were not too large to enter the gel. Other studies reporting about the EMSA of QD/DNA complexes have evidenced that QDs completely inhibited the DNA from moving towards the positive electrode when the QDs/DNA was just at or excessive stoichiometry [24]. The phenomenon was accounted to the negative charges of DNA that were counteracted by the positively charged QDs or to the large size of the newly formed complexes unable to enter the gel. This problem was not experienced during the EMSA of SslA_341-925_streptavidin and biotinylated quantum dots; the difference in the migration pattern of these moieties proves a successful binding reaction. Another work implying the fusion of the same streptavidin domain to the S-layer protein of *Bacillus stearothermophylus* ATCC 12980 (SbsC) could not determine the functionality of the fused streptavidin domain despite testing with EMSA of biotinylated DNA and the S-layer/streptavidin chimeric protein [23]. The reasons suggested included the inability of the fusion protein to bind biotinylated molecules, the large size of the DNA molecules (4300 bps long) in comparison to the fusion protein, and the tetrameric structure of streptavidin with the biotin binding site lying at the interface of the 4 subunits [25,26].

A further prove of the preserved biotin binding ability of the SslA_341-925_streptavidin are the TEM images of the negatively stained, patterned protein layers, which reveal that the biotinylated quantum dots are bound to the protein template. On tubular structures, a more accentuated marginal deposition of quantum dots is observed. The substrate onto which the fusion protein has been immobilised might also play a role. Due to the O_2_ plasma treatment step, the carbon coated Cu grid surface can be regarded as being more hydrophilic, therefore the most important driving force for the protein adsorption are the long range electrostatic interactions occurring between the positively charged sites on the fusion protein and the negative charges of the substrate. As a result, the SslA_341-925_streptavidin adsorbs in a way such that the streptavidin moiety remains exposed. However when the protein lattice folds into tube like structures, the streptavidin domain becomes buried and no longer accessible to the biotinylated quantum dots. Therefore no particles are bound onto these protein regions. Marginal deposition is possible though, either due to the fact that the streptavidin domains are located at the edges of the protein sheets, or because during folding, tubes do not close completely, but the layer edges simply meet each other. It is further interesting to note that only the S-layer part of the fusion protein did not fold into tube like structures [17]. Coupling the S-layer to the streptavidin domain indeed results in extension of the protein. This extended protein structure allows the adoption of a certain curvature when assembled into protein sheets.

In conclusion, the SslA_341-925_streptavidin fusion protein self-assembles into uniform, coherent layers in solution and on silicon wafers. This remarkable property facilitates the formation of a two-dimensional protein array with repetitive features in the nanometer range that exposes streptavidin. Due to the high affinity between streptavidin and biotin and the fact that nearly each molecule can be biotinylated, the SslA_341-925_streptavidin protein template can be considered a functional biomolecular matrix for immobilizing biotinylated molecules in a controlled manner.

## 4. Materials and Methods

### 4.1. Bacterial Strains and Growth Conditions

*Sporosarcina ureae* ATCC 13881 cells were grown at 30 °C in S ureae growth medium I (17.01 g Na_3_PO_4_·12H_2_O, 1.28 g glucose, 1.0 g yeast extract, 10 g peptone, 5.28 g (NH_4_)_2_SO_4_) while continuously agitating at 200 rpm until OD_600_ = 1.5, which is within the logarithmic growth phase.

*E. coli* cells were grown in liquid at 37 °C under continuous agitation at 140 rpm. For selection of bacterial transformants, ampicillin was added to the media (final concentration 110 μg/mL). For bacterial gene expression in *E. coli* expression strains, cultures were grown until an optical density OD_600_ = 0.4 and were afterwards induced with isopropyl β-D-1 thiogalactopyranoside (IPTG) (1 mM final concentration).

### 4.2. Cloning of SslA_341-925_streptavidin

The SslA_341-925_streptavidin was created by overlap extension PCR. In a first step, the DNA sequence encoding SslA_341-925_ was amplified using the oligonucleotide primers A_for_ 5′-CGCGGCCATATGGGCGTTAAAAAAGCAGGAAT and C_rev_ 5′-GTACCAGGTGCCGGTGATGCCCGAACTAATAACTAATGCATTTGCAGTTG. Next, streptavidin (aa 16-133) was amplified by PCR with primers B_for_ 5′-ATGCATTAGTTATTAGTTCGGGCATCACCGGCACCTGGTA and D_rev_ 5′-CACTAGCTCGAGCACCTTGGTGAAGGTGTCGTGG. The two amplified DNA fragments were mixed in a third PCR reaction for obtaining the chimeric gene SslA_341-925_streptavidin, which was then cloned into the pET46 Ek/Lic (Novagen, Darmstadt, Germany) vector using the Ek/Lic cloning strategy. The recombinant plasmid pET46Ek/Lic-SslA_341-925_streptavidin was transformed by electroporation with a Gene Pulser II (voltage: 2.5 kV; capacity: 25 μF; resistance: 200 Ω, BIORAD, München, Germany) into *E. coli* Nova Blue Giga Singles (Novagen, Darmstadt, Germany) competent cells as a non-expression host.

### 4.3. Expression, Isolation, and Purification of SslA_341-925_streptavidin

For heterologous expression, the pET46Ek/Lic-SslA_341-925_streptavidin plasmid was transformed into *E. coli* Rosetta Blue (DE3) cells. For expression, 0.5 L of Luria-Bertani (LB) medium was inoculated with 5 mL of the LB-grown preculture of *E. coli* Rosetta Blue (DE3). The cultures were grown at 37 °C until an optical density OD_600_ = 0.4, and then recombinant gene expression was induced with 1 mM IPTG. 5 h after the induction of expression, cells were harvested by centrifugation (10,000× *g* for 10 min), washed twice with ddH_2_O, and the pellet resuspended in 20 mL buffer consisting of 20 mM Tris/HCl (pH = 7.9), 0.5 M NaCl, 5 mM imidazol (AppliChem, Darmstadt, Germany), 10 mg lysozyme (New England Biolabs, Frankfurt, Germany) and 1 mM AEBSF (AppliChem, Darmstadt, Germany). After incubation at 30 °C for 2 h under continuous shaking at 300 rpm, the cell suspension was chilled on ice for 15 min. Then, Triton X-100 (Sigma-Aldrich, München, Germany) was added in a final concentration of 0.5% (*v*/*v*) and the cells were broken up by sonication (Bandelin Sonopuls UW 2070, Berlin, Germany). Following sonication, RNase A and DNase I (Roth, Karlsruhe, Germany) were given to the mixture and incubated at 30 °C for 15 min under continuous shaking followed by centrifugation at 13,000× *g* for 10 min in order to separate the insoluble cell fraction. The supernatant was applied to a Ni affinity chromatography purification column (Macherey-Nagel, Düren, Germany) and purified as described in [19]. Five elution fractions were collected and immediately dialysed against ultrapure water at 4 °C overnight. Expression was controlled by SDS-PAGE of cell extracts performed according to Laemmli (1970) [27].

### 4.4. In Vitro Recrystallisation in Solution and on a Silicon Wafer of SslA_341-925_streptavidin

In vitro recrystallisation experiments were conducted in solution and on a Si substrate. In the first case, the purified SslA_341-925_streptavidin fusion protein (at a concentration of 1 mg/mL) was monomerized with 5 M GuHCl which was subsequently removed by dialysis against distilled water for 3 h at 4 °C. After dialysis, the protein solution was centrifuged at 14,000× *g* for 10 min and the supernatant containing the SslA_341-925_streptavidin monomers was diluted to 0.1 mg/mL with 0.5 mM Tris/HCl buffer pH = 9 and 0.1 mM CaCl_2_ until a total volume of 10 mL. 5 mL of this mixture was pipetted into a cell culture well and the fusion protein was allowed to recrystallize for 48 h. After recrystallisation, protein samples were taken out and prepared for TEM.

For in vitro recrystallisation on a silicon substrate, 0.5 mg of the purified SslA_341-925_streptavidin protein was dissolved in 5 M GuHCl, and then 1/5 of the solution was dialysed against Tris/HCl buffer (pH = 3) for 2 h at 4 °C in the presence of an APTES-functionalised silicon substrate. After dialysis, the silicon substrate was washed with distilled water, dried on air, and the self-assembly structures analysed by AFM.

### 4.5. Preparation of APTES Functionalised Silicon Substrates

Silicon pieces were prepared according to [19].

### 4.6. Investigation of Biotin-Binding Ability of SslA_341-925_streptavidin

Qdot Biotin Conjugates (CdSe/ZnS coated with a polymer shell and conjugated to biotin) in H_2_O were purchased from Invitrogen, Carlsbad, CA, USA and were used as received. According to the manufacturer’s specifications, the size of the Qdot Biotin conjugate is 10–12 nm and the emission wavelength maximum is near 605 nm.

For binding of biotinylated quantum dots to the SslA_341-925_streptavidin monomers, 1 mg of purified fusion protein was monomerized with 5 M GuHCl followed by removal of the chemical by a dialysis step against distilled water for 2 h at 4 °C. After dialysis, the protein solution was centrifuged for 10 min at 14,000× *g* and the supernatant containing the fusion protein monomers was mixed with quantum dots. Mixtures containing different amounts of the fusion protein (5, 15 and 50 μg), 20 mM quantum dots (CdSe/ZnS coupled to biotin, purchased from Invitrogen), and 10 μL 20 mM KHPO_4_ (pH = 6.4) were incubated for 2 h at room temperature, then separated on a 1% agarose gel at 80 V for 2 h.

For the binding studies, at first chimeric protein layers were formed by in vitro recrystallisation in solution and afterwards were immobilised onto plasma treated carbon-coated copper grids and allowed to adsorb for 1 h. Biotinylated quantum dots (at a concentration of 0.02 nM in 20 mM KHPO_4_ buffer) were allowed to adsorb for 60 min onto the S-layer sheets. Excess particles were washed away with several drops of TBS buffer (0.2M Tris/HCl, pH = 7.4, 1.37 M NaCl) and the samples were additionally stained with 2% uranyl formiate for 20 s.

### 4.7. Atomic Force Microscopy

The measurements were performed in tapping mode in air using the Extended Multimode AFM with Nanoscope IIIa controller system Digital Instruments, Inc. Veeco Metrology, Santa Barbara, CA, USA). Silicon tips that were 130 µm long were used for imaging. The images were analyzed using the software WsxM (Nanotech Electronica S.L., Madrid, Spain).

### 4.8. Transmission Electron Microscopy

Self-assembly products were prepared for imaging as follows: formvar/carbon films on copper grids (PLANO, Wetzlar, Germany) were plasma treated (O_2_ SPI Plasma Prep II, West Chester, USA) for 10 s in order to make them hydrophilic. Directly thereafter, 10 µL of fusion protein solution was applied onto the grid and left to adsorb for 1 h. Afterwards, the grid was washed with distilled water and the protein stained with 7 µL of 2% uranyl formiate. After staining, the whole grid was blotted with filter paper and dried on air. Transmission electron micrographs were obtained on a Zeiss LIBRA200FEG (Oberkochen, Germany) using 200 kV acceleration voltage.
